# Selection of 3D-Printing Optimal Parameters via MCDM for Maximizing the Thermomechanical Response of TPU and PEEK

**DOI:** 10.3390/polym18121468

**Published:** 2026-06-11

**Authors:** Jorge Crespo-Sánchez, Daniel Fernández, Claudia Solek, Jorge Ayllón, Sergio Fuentes del Toro, Ana María Camacho, Álvaro Rodríguez-Prieto

**Affiliations:** 1 Department of Manufacturing Engineering, Universidad Nacional de Educación a Distancia (UNED), C. Juan del Rosal, 12, 28040 Madrid, Spain; dfernande146@alumno.uned.es (D.F.); csolek@ind.uned.es (C.S.); jorge.ayllon@ind.uned.es (J.A.); amcamacho@ind.uned.es (A.M.C.); alvaro.rodriguez@ind.uned.es (Á.R.-P.); 2 Department of Mechanical Engineering, Universidad Carlos III de Madrid, Avda. De la Universidad, 30, 28912 Leganes, Spain; sfuentes@ing.uc3m.es

**Keywords:** fused filament fabrication, dynamic mechanical analysis (DMA), ANOVA, entropy weight method, design of experiments

## Abstract

The optimization of Fused Filament Fabrication (FFF) process parameters is commonly performed using room-temperature mechanical properties as the main decision criteria, while the temperature-dependent thermomechanical response of printed polymers is often not explicitly considered. This limitation is relevant for functional components intended to operate above room temperature, where stiffness retention and viscoelastic behavior may strongly affect service performance. This work proposes an experimental–statistical framework for selecting FFF parameters by integrating Design of Experiments (DoE), tensile testing, dynamic mechanical analysis (DMA), Analysis of Variance (ANOVA), the Entropy Weight Method (EWM) and the VIKOR method. Two materials with contrasting thermomechanical behavior were investigated: a high-performance semicrystalline polymer, Z-PEEK, and an elastomeric thermoplastic, TPU 95A. For each material, a DoE was defined to evaluate the influence of key printing parameters, and the manufactured specimens were characterized in terms of maximum tensile force, maximum deformation and storage modulus at selected temperatures. The ANOVA results showed a material-dependent influence of the processing parameters, with thermally driven parameters being especially relevant for Z-PEEK and deposition-related parameters having a stronger influence on TPU 95A. The EWM–VIKOR analysis identified the optimal Z-PEEK configuration as 400 °C extrusion temperature, 200 °C build plate temperature and 150 °C chamber temperature, whereas the optimal TPU 95A configuration corresponded to 225 °C extrusion temperature, 0.10 mm layer height, 50 mm/s printing speed and 80 °C build plate temperature. Overall, the results demonstrate that incorporating DMA-derived thermomechanical indicators into MCDM-based optimization provides a more application-oriented basis for FFF parameter selection than approaches based only on room-temperature mechanical properties.

## 1. Introduction

Additive manufacturing (AM), also referred to as 3D printing, enables the fabrication of three-dimensional components through a layer-by-layer process based on a digital model. According to ISO/ASTM 52900:2021 [1], AM is defined as “the process of joining materials to make parts from 3D model data, usually layer upon layer”. Since its early development in the 1960s [2], AM has experienced a profound technological evolution, transitioning from a set of techniques primarily oriented toward rapid prototyping into a mature manufacturing paradigm adopted across a wide range of industrial sectors. Current applications span areas such as medicine [3], construction [4], automotive engineering [5] and textile and apparel industries [6], where AM provides significant added value in terms of design freedom, functional integration, and customization capabilities [7].

The rapid growth of AM adoption is reflected in the strong expansion of its global market value, which increased from approximately USD 6.1 billion in 2016 [8] to a projected value of USD 196.8 billion by 2035 [9]. This sustained growth is largely driven by the intrinsic advantages of AM technologies when compared to conventional subtractive manufacturing processes. These advantages include the ability to produce highly customized and geometrically complex parts, reduced manufacturing lead times, cost-effective production start-up, minimized material waste, and access to a continuously expanding portfolio of materials—including polymers, metals, composites, and construction materials. Moreover, significant advances in process control, material science, and computational modeling have further enhanced the reliability, repeatability, and performance of additively manufactured components, thereby enabling their use in functional and structural applications.

Several classification frameworks have been proposed to categorize AM techniques [10], with the most widely accepted being that established by ASTM Standard F2792 [11]. This classification groups AM technologies into seven main categories: Powder Bed Fusion (PBF) [12], Directed Energy Deposition (DED) [13], Material Extrusion (ME) [14], Vat Photopolymerization (VAT) [15], Binder Jetting (BJ) [16], Material Jetting (MJ) [17] and Sheet Lamination (SL) [18]. These categories encompass a broad spectrum of processing principles and material systems, including metals, polymers and cementitious materials.

Among these technologies, this work focuses on Material Extrusion, and specifically on polymeric Fused Filament Fabrication (FFF), as defined by ISO/ASTM 52900 [1]. FFF is one of the most extensively used AM techniques due to its relative simplicity, low equipment and operating costs, and its ability to process a wide range of materials, with development predominantly focused on thermoplastic polymers.

A typical polymeric FFF process consists of feeding a thermoplastic filament from a filament spool into a heated extruder with a defined nozzle diameter [19].

The filament, which may be amorphous or semicrystalline depending on the polymer used [20], is then heated above its melting or softening temperature and subsequently extruded through the nozzle in a molten or viscoelastic state for material deposition [21]. Guided by the digital model data of the desired part, the nozzle moves in the horizontal plane while depositing the molten material onto a printing bed. Upon deposition, the material rapidly solidifies and adheres to the build platform in the case of the first layer, and to the previously deposited material for subsequent layers, thereby forming each layer of the printed component [22]. This sequence is repeated layer by layer until the complete part is manufactured.

Modern FFF systems frequently incorporate dual-extrusion configurations, allowing the use of different materials during the same printing process. Typically, one extruder is dedicated to the structural material, while the second is employed to deposit a support material, enabling the fabrication of complex geometries, overhangs, and internal cavities that would otherwise be unprintable [23].

The printing bed and the build chamber are commonly heated or conditioned using surface treatments or adhesives to enhance adhesion between the molten polymer and the build platform. Additionally, temperature control of the bed and chamber contributes to reducing thermal gradients between the extrusion temperature and the ambient environment, thereby mitigating thermally induced defects such as warpage, residual stress, and dimensional distortions (e.g., spring-in effects) [24,25].

However, the final quality of the printed component, as well as its mechanical and thermal response, strongly depends on both the selected printing parameters and the material employed, making their optimization essential to achieve optimal performance. These parameters have been extensively investigated for a wide range of polymeric materials and printing systems, including high-performance thermoplastics such as PEEK and PEKK [26], as well as more widely used polymers such as PLA [27,28], PBS [29], and TPU [30].

Although the industrial adoption of AM has increased significantly, the central challenge addressed in this work is not the expansion of AM itself, but the selection of suitable FFF process parameters when multiple performance criteria must be considered simultaneously. In polymeric FFF, process parameters such as extrusion temperature, build plate temperature, chamber temperature, layer height, and printing speed affect the thermal history of the deposited material, interlayer consolidation, mechanical strength, deformation response, and stiffness retention under temperature.

Consequently, the optimization of FFF components is inherently multi-objective. Parameter combinations that improve one response, such as maximum tensile force, may not necessarily provide the best performance in terms of deformation control or thermomechanical stability. This is particularly relevant for functional polymer components operating above room temperature, where storage modulus and viscoelastic behavior may vary substantially with temperature. Therefore, MCDM methods are useful for identifying compromise solutions among competing mechanical and thermomechanical objectives.

Nevertheless, a critical review of the existing literature reveals that most statistical analyses and optimization approaches follow different methodological strategies. For instance, studies such as [31,32] focused on the application of Response Surface Methodology (RSM) to identify optimal combinations of process parameters—such as raster angle, infill percentage, and extrusion temperature—aimed at maximizing mechanical performance under standard testing conditions.

In contrast, works such as [33,34] explored the influence of multiple processing parameters on the mechanical response of FFF parts, emphasizing the interaction effects between variables and their impact on tensile and flexural properties. Additionally, other contributions, including [35,36], adopted multi-response optimization strategies to simultaneously evaluate different mechanical indicators, highlighting the complexity of process–property relationships in FFF.

However, despite these differences in scope and methodological approach, most of these studies remain focused on optimizing mechanical performance under standard conditions, without incorporating the influence of service or operating temperature as a primary decision criterion. This limitation is particularly relevant for FFF-manufactured polymeric components intended for functional applications, where stiffness retention, viscoelastic response, and thermomechanical stability under service-like thermal conditions may strongly affect final performance.

The specific research gap addressed in this work is the limited integration of temperature-dependent thermomechanical properties into MCDM-based optimization strategies for FFF polymers. While ANOVA, EWM, and VIKOR are well-established methods, their combined use is here implemented within an application-oriented framework that integrates tensile response and DMA-derived storage modulus values for the selection of FFF process parameters. Therefore, the novelty of this study does not lie in the individual use of these methods, but in their combined application to optimize FFF printing parameters considering both mechanical performance and temperature-dependent stiffness. In this context, the present work adopts a Design of Experiments (DoE) framework to systematically investigate the influence of key FFF printing parameters on the mechanical and thermal response of additively manufactured polymeric components. Unlike conventional optimization studies that primarily focus on room-temperature mechanical performance, this work explicitly incorporates the temperature-dependent behavior of printed parts as a core response variable. This approach enables the identification of statistically significant parameter effects and interactions governing not only strength and stiffness, but also their evolution under thermally relevant service conditions.

Furthermore, the proposed DoE-based methodology is applied to different sets of printing parameters and two polymeric materials with markedly contrasting thermomechanical behaviors, namely polyether etherketone (PEEK) and thermoplastic polyurethane (TPU 95A). These materials were intentionally selected to span a wide spectrum of stiffness, viscoelasticity, and temperature sensitivity, allowing for a comprehensive evaluation of the robustness and generality of the proposed framework. By simultaneously addressing a high-performance semicrystalline thermoplastic and a hyperelastic material, this study moves beyond material-specific optimization toward a unified and transferable experimental–statistical methodology.

Overall, this work provides an application-oriented MCDM framework for selecting FFF process parameters based on both mechanical and thermomechanical performance. The proposed multi-material and multi-parameter strategy contributes to a more realistic assessment of FFF-manufactured polymeric components by considering not only room-temperature behavior but also stiffness retention under relevant service-temperature conditions.

The article is organized as follows. Section 2 describes the materials, printing systems, experimental design, mechanical and thermomechanical testing procedures and statistical–MCDM methodology. Section 3 presents and discusses the experimental results, ANOVA analysis and MCDM-based selection of the optimal printing parameters. Finally, Section 4 summarizes the main conclusions, limitations and future research directions.

## 2. Materials and Methods

**Figure 1:**
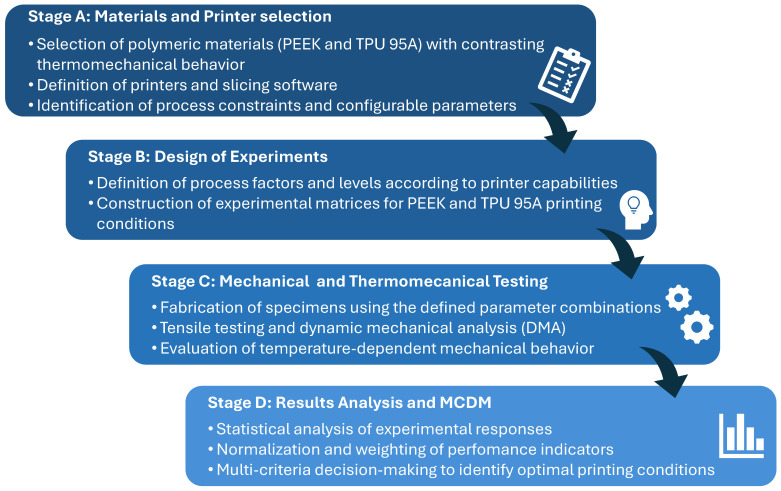
Schematic representation of the multi-stage experimental and analytical framework adopted in this study, from DoE formulation to MCDM-based optimization.

Figure 1 illustrates the methodological workflow developed in this study. The proposed framework is divided into four sequential stages: (A) materials and printing platform selection, (B) Design of Experiments (DoE) formulation, (C) mechanical and thermomechanical testing of the fabricated specimens, and (D) results analysis and selection of optimal parameters through a MCDM methodology.

### 2.1. Materials and Printer Selection (Stage A)

In this work, two fundamentally different polymeric materials were investigated, each processed using distinct FFF printing systems and independent slicing software environments. This approach was adopted with the explicit objective of demonstrating the robustness, versatility, and general applicability of the proposed experimental methodology. By considering different material–machine–software combinations, the study ensures that the obtained results are not constrained to a specific setup, thereby reinforcing the methodological validity of the approach.

Among high-performance polymers, PEEK has become a preferred material for the manufacturing of advanced engineering components due to its outstanding physicochemical properties, including excellent thermal stability, high mechanical strength, intrinsic self-lubrication, and biocompatibility. These characteristics have enabled its widespread adoption in demanding applications, particularly in biomedical engineering, where PEEK has demonstrated significant clinical and technological value [37]. According to reports from the U.S. Food and Drug Administration (FDA), nearly 90% of lumbar spinal cages cleared in 2018 were manufactured from PEEK [38], highlighting its relevance in industrial and biomedical applications.

From a processing perspective, it has been demonstrated that achieving a balanced distribution of amorphous and crystalline phases, together with high interlayer adhesion, is essential for producing mechanically robust PEEK components via FFF. Effective interlayer bonding is typically associated with minimized interlayer gaps, reduced visibility of deposited filaments, and improved continuity between layers, which enhance load transfer across the build direction [39]. Prior studies have examined the influence of FFF process parameters—such as nozzle temperature, chamber temperature, printing speed, and raster angle—on the mechanical performance of PEEK components. Under optimized conditions, FFF-printed PEEK parts can reach mechanical properties comparable to injection-molded counterparts, with reported values of approximately 96 MPa tensile strength, 31% strain at break, and 3.7 GPa flexural modulus [40].

Despite these advances, a critical limitation remains in the literature: the thermomechanical response of FFF-printed PEEK components has not been systematically addressed. This gap is particularly relevant given that many PEEK applications—especially in biomedical, aerospace, and industrial environments—involve elevated or controlled temperature conditions, requiring a clear understanding of property evolution with temperature. As explained in the introduction, this gap motivates the thermomechanical analysis conducted in this work.

Based on this context, a high-performance thermoplastic, Z-PEEK (Zortrax, Olsztyn, Poland) [41], with a filament diameter of 1.75 mm, was selected. Z-PEEK is a biocompatible, semicrystalline polymer characterized by high stiffness, a high melting temperature (with recommended extrusion temperatures above 375 °C), and a glass transition temperature of approximately 143 °C, as determined by differential scanning calorimetry (DSC). Additionally, it exhibits high resistance to abrasion and corrosion, making it suitable for demanding industrial applications. All Z-PEEK specimens were manufactured using a Zortrax Endureal FFF printer (Zortrax, Poland) [42] and processed with *Z-Suite v2.32.0*^®^ [43]. This system operates within a closed software environment, where only proprietary files can be used, limiting the number of adjustable printing parameters. This constraint provides a relevant scenario for evaluating the proposed DoE methodology under restricted parameter configurability.

In contrast to high-performance thermoplastics, thermoplastic polyurethanes (TPU) represent a class of elastomeric polymers widely used in additive manufacturing due to their high flexibility, resilience, and abrasion resistance. TPU materials are characterized by a segmented microstructure composed of soft and hard domains, which results in pronounced viscoelastic behavior and strong temperature dependence of their mechanical properties. These features make TPU particularly suitable for applications requiring energy absorption, damping, and compliance, such as wearable devices, soft robotics, and protective components.

From a processing perspective, the FFF fabrication of TPU presents specific challenges related to its low stiffness and high deformability, which can affect material feeding, dimensional accuracy, and interlayer bonding. Several studies have investigated the influence of key printing parameters—such as extrusion temperature, printing speed, and layer height—on the mechanical response and surface quality of TPU parts, highlighting the sensitivity of elastomeric materials to processing conditions [44]. However, similar to the case of PEEK, most existing works focus primarily on room-temperature mechanical properties, with limited attention paid to the thermomechanical response and its evolution with temperature.

Given the intrinsic viscoelastic nature of TPU, understanding the variation in mechanical properties as a function of temperature is essential for reliable design and application. In particular, parameters such as the storage modulus (E′) and loss factor (tan δ) play a key role in describing stiffness degradation and energy dissipation mechanisms. Therefore, the inclusion of TPU in this study provides a complementary perspective to high-performance polymers, enabling the evaluation of the proposed methodology across materials with markedly different thermomechanical behaviors.

On the other hand, a thermoplastic elastomer, TPU 95A supplied by Ultimaker (Utrecht, Holland) [45], with a filament diameter of 2.85 mm, was selected to represent a material with significantly lower stiffness, reduced crystallinity, and pronounced viscoelastic behavior. The TPU specimens were printed using an Ultimaker S5 printer (Utretch, Holland) [46], for which recommended extrusion temperatures range between 210 °C and 240 °C and printing speeds between 20 and 80 mm/min. In this case, *Ultimaker Cura v5.8.1*^®^ [47] was used for slicing and print preparation. Unlike the *Z-Suite v2.32.0*, *Ultimaker Cura v5.8.1*^®^ offers extensive parameter configurability, enabling the selection of a broader set of process variables. As a result, four printing parameters were considered in the DoE analysis for TPU, compared to three parameters in the Z-PEEK case.

In addition to their distinct thermomechanical behavior, the selection of PEEK and TPU in this study is also motivated by their thermoplastic nature, which enables repairability, recyclability, and reprocessability. These characteristics make them attractive alternatives to conventional materials such as nitrile butadiene rubber (NBR) or epoxy-based resins, particularly in applications where sustainability, material efficiency, and lifecycle extension are increasingly relevant.

Furthermore, as mentioned, both materials can be processed using (FFF), a highly versatile and widely accessible AM technology. This contributes to increased manufacturing flexibility, reduced production costs, and improved availability of components, especially in decentralized or on-demand production scenarios.

Finally, due to their markedly different mechanical and thermomechanical properties, PEEK and TPU can be considered complementary materials. Their combined use in multi-material or hybrid structures opens the possibility of designing advanced components with tailored functionalities, such as vibration damping or impact absorption, where stiffness and flexibility must be simultaneously optimized.

The main properties of Z-PEEK and TPU 95A filaments are summarized in Table 1, as provided by the respective manufacturers in their technical data sheets.

### 2.2. Design of Experiments (DoE) (Stage B)

Design of Experiments (DoE) is a statistical and methodological framework that allows the systematic evaluation of the influence of multiple process parameters on the resulting properties of manufactured components while significantly reducing the number of required experiments. In FFF, where numerous controllable variables may influence the mechanical and thermal performance of the printed parts, DoE provides an efficient strategy to explore the parameter space and identify statistically significant factors. By defining factors and levels in a structured experimental matrix, the method enables the quantification of parameter effects and interactions, facilitating the identification of optimal processing conditions. In this work, a DoE approach is adopted to evaluate how selected printing parameters affect the mechanical and thermomechanical response of the manufactured specimens.

In this work, two different DoE schemes, constructed based on modified Taguchi orthogonal arrays, were implemented to evaluate the influence of selected printing parameters on the mechanical and thermomechanical response of the fabricated parts. The configuration of each DoE, in addition to its corresponding factors and levels, is presented in the following sections.

#### 2.2.1. PEEK

The DoE for Z-PEEK was defined considering both the processing limitations of the printing system and the thermally driven nature of the material. Unlike open FFF platforms, the Zortrax Endureal system operates within a closed software environment, which significantly restricts the number of adjustable parameters. As a result, only three process variables could be modified: extrusion temperature (ET), build plate temperature (PT), and chamber temperature (CT).

These parameters were selected due to their direct influence on the thermal history of the material during deposition, which is known to be a critical factor in the processing of semicrystalline polymers such as PEEK. In particular, the ET governs melt viscosity and interlayer diffusion, while the PT and CT control the cooling rate, thermal gradients, and crystallization kinetics during solidification.

Each parameter was evaluated at three levels, defined based on both manufacturer recommendations and process feasibility. The ET was varied between 375 °C (recommended by the supplier), 400 °C and 425 °C, allowing the assessment of rheological behavior within the operational processing window. The CT was set at 125 °C, 150 °C and 170 °C, covering a range around the glass transition temperature of PEEK (~143 °C), where molecular mobility and stress relaxation phenomena are expected to play a significant role. Finally, the PT was varied between 140 °C, 170 °C and 200 °C to evaluate its effect on interlayer adhesion and thermal gradient reduction during the initial stages of deposition. The layer height was intentionally kept constant due to the constraints imposed by the closed software environment, which restricts the independent adjustment of this parameter and ensures process consistency across all experimental conditions.

A full factorial design with three factors and three levels (3^3^) would result in 27 experimental combinations. However, due to physical constraints of the system, not all combinations were feasible. Specifically, configurations in which the CT exceeded the PT were excluded, as they lead to unstable thermal conditions during printing. As a result, a total of 21 valid experimental conditions were defined and executed.

The exclusion of infeasible Z-PEEK conditions implies that the final experimental matrix is not a fully balanced 3^3^ factorial design. Consequently, the ANOVA and MCDM results for Z-PEEK must be interpreted within the constrained feasible domain imposed by the printing platform. Although this restriction may influence the estimation of factor effects, it reflects the actual operating window of the machine and therefore preserves the practical relevance of the optimization.

The complete set of process parameters and experimental configurations for Z-PEEK DoE are presented in Table 2 and Table 3.

#### 2.2.2. TPU 95A

In contrast, for the TPU 95A material, the *Ultimaker Cura v5.8.1*^®^ [47] allows a significantly higher level of parameter configurability. As a result, four printing parameters were considered as experimental factors in the corresponding DoE. The extrusion temperature was varied between 225 °C, 240 °C and 255 °C, while the layer height (LH) was set at 0.10 mm, 0.15 mm and 0.20 mm. Additionally, the printing speed (PS) was evaluated at 20 mm/s, 50 mm/s and 80 mm/s, and the build plate temperature was varied between 60 °C, 70 °C and 80 °C. This expanded parameter space enables a broader evaluation of how processing conditions influence the mechanical and thermomechanical behavior of TPU specimens. These parameters are shown in Table 4.

Considering that each factor was evaluated at three levels, a full-factorial design would require 3^4^ = 81 experimental combinations, which would substantially increase the experimental effort and manufacturing time. To address this limitation while preserving the statistical robustness of the analysis, a Taguchi orthogonal array was adopted to reduce the number of experimental runs. This approach maintains the orthogonality of the experimental matrix, allowing the independent evaluation of factor effects while significantly decreasing the number of required experiments. The resulting L27 Taguchi experimental design is summarized in Table 5 and ensures an efficient exploration of the parameter space while maintaining statistical reliability.

### 2.3. Mechanical and Thermomechanical Testing (Stage C)

**Figure 2:**
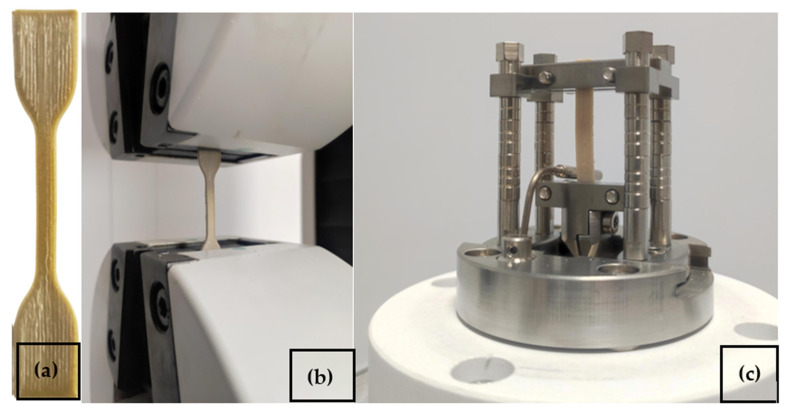
(**a**) Geometry of the ISO 37 Type 2 tensile specimen; (**b**) specimen positioning in the universal testing machine during tensile testing; (**c**) DMA experimental setup without the upper clamping enclosure to enable direct temperature control.

For the mechanical characterization, tensile specimens were manufactured in accordance with ISO 37 Type 2 [48] and ISO 527 Type 5A [49] standards as shown in Figure 2a, both of which provide equivalent geometrical dimensions for tensile testing. These specimens were tested under uniaxial loading conditions using a universal testing machine (Hoytom HM-D, 100 kN, Leioa, Spain) to evaluate the influence of the selected printing parameters on the tensile behavior of the fabricated parts as shown in Figure 2a.

In accordance with the standards, three repetitions were performed for each experimental condition, and the reported results correspond to the average values of maximum force and displacement. The tensile tests were conducted at a crosshead speed of 1 mm/min for Z-PEEK and 10 mm/min for TPU 95A, in order to account for the different mechanical behavior of the materials and to ensure stable loading conditions while minimizing inertia-related effects. In accordance with the standards, three repetitions were performed for each experimental condition, and the reported results correspond to the average values of maximum force and displacement.

Regarding the printing strategy for the tensile specimens, the raster orientation was alternated orthogonally between successive layers. This configuration was selected based on previous findings [50], where it was demonstrated that cross-layer raster orientations lead to a more isotropic tensile response, with minimal variation in mechanical properties compared to other printing strategies. The scheme used in the tensile test is shown in Figure 2b.

For thermomechanical characterization, rectangular specimens with dimensions of 30 × 4 × 1.25 mm were tested using a DMA-1 dynamic mechanical analyzer (Mettler Toledo, Cornellà del Llobregat, Spain) in tensile mode, as shown in Figure 2c. The tests were performed at a frequency of 1 Hz using a heating rate of 3 °C/min to ensure a homogeneous temperature distribution within the specimen, as recommended by ISO 6721 [51]. For TPU 95A, the temperature range was set from 25 to 170 °C, whereas for Z-PEEK, the analysis was extended from 25 to 225 °C, reflecting its higher thermal resistance. Once the maximum test temperature was reached, the specimens were maintained at that temperature for 10 min in order to stabilize the thermomechanical response. For each experimental condition, three independent specimens were manufactured and tested. In order to minimize process-related variability, the three specimens corresponding to each condition were printed within the same printing batch, using the same material, machine setup, and processing parameters. This strategy was adopted to reduce the influence of external sources of variability, such as machine recalibration, environmental changes, filament condition, or differences between printing sessions. The reported mechanical and thermomechanical values correspond to the mean value obtained from the three repetitions, and the associated standard deviation was calculated to evaluate the experimental dispersion and repeatability of the measurements.

For the thermomechanical analysis, the storage modulus (E′) was evaluated at selected temperature points in order to characterize the evolution of material stiffness with temperature. This modulus represents the elastic component of the viscoelastic response, providing a direct measure of the material stiffness as a function of temperature. This analysis enables the identification of stiffness degradation and the thermal stability of the printed materials under service-like conditions.

In the case of TPU 95A, E′ was extracted at 25 °C, 75 °C, 100 °C, and 150 °C, while for Z-PEEK, the analysis was extended to 25 °C, 75 °C, 100 °C, 150 °C, and 200 °C.

In addition, the maximum values of tan δ (loss factor) were determined for each specimen. The parameter tan δ, defined as the ratio between the loss modulus (E″) and the storage modulus (E′), is a key indicator of the material’s viscoelastic behavior and energy dissipation capacity. The peak of the tan δ curve is commonly associated with the glass transition temperature (T_g_), corresponding to the transition from a predominantly elastic (glassy) state to a more viscoelastic or rubber-like behavior. Therefore, the analysis of tan δ provides valuable insight into the temperature-dependent transition mechanisms of the materials and their suitability for applications involving thermal loading.

Finally, these thermomechanical indicators, shown in Table 6 and Table 7, were used as response variables in the subsequent Analysis of Variance (ANOVA) and Multi-Criteria Decision-Making (MCDM) analysis, enabling the evaluation of the influence of printing parameters on the temperature-dependent mechanical performance of the materials.

### 2.4. Statistical Analysis and MCDM (Stage D)

To comprehensively evaluate the influence of the selected printing parameters and identify optimal processing conditions, a two-stage analysis framework was adopted. First, a statistical analysis based on Analysis of Variance (ANOVA) [52] was performed to quantify the significance and contribution of each factor. Subsequently, a MCDM approach was applied to determine the optimal parameter combination by simultaneously considering multiple performance indicators.

#### 2.4.1. ANOVA

Analysis of Variance (ANOVA) is a statistical method used to quantify the influence of input factors on the variability of a given response variable. In the context of this study, ANOVA was applied to evaluate the statistical significance of the selected FFF process parameters and to identify the relative contribution of each factor to the observed mechanical and thermomechanical responses.

The total variability of the experimental data is expressed through the total sum of squares (SST), defined by Equation (1):

(1) $$\;SST=\sum_{i=1}^{N}(y_{i}-\overset{-}{y}{)}^{2}$$

where $y_{i}$ represents the individual experimental observations, $\overset{-}{y}$ is the overall mean of the dataset, and N is the total number of observations.

The contribution of each factor j to the total variability is quantified by its corresponding sum of squares $SS_{j}$. The mean square associated with each factor is then calculated as Equation (2):

(2) $$MS_{j}=\frac{SS_{j}}{df_{j}}$$

where $df_{j}$ denotes the degrees of freedom associated with factor j.

The statistical significance of each factor is evaluated using the F-ratio, given by Equation (3):

(3) $$F_{j}=\frac{MS_{j}}{MS_{e}}$$

Higher values of $F_{j}$ indicate a greater influence of the corresponding factor on the response variable. In addition, the *p*-value, which is defined in Equation (4) and associated with each factor, is obtained from the cumulative distribution function of the F-distribution, considering the calculated $F_{j}$ value and the corresponding degrees of freedom. It is used to assess the statistical relevance of each factor. In this work, a significance level of α=0.05 (95% confidence level) was adopted. Therefore, factors with *p*-values lower than 0.05 are considered statistically significant.

(4) $$p_{j}=P(F_{df_{j},\;df_{e}}\geq F_{j})$$

where $F_{df_{j},\;df_{e}}$ denotes the F-distributed random variable with $df_{j}$ and $df_{e}$ degrees of freedom.

To further quantify the relative importance of each parameter, the percentage contribution $\left(\%\;Contrib.\right)$ was calculated as Equation (5):

(5) $$\%\;Contrib.=\frac{SS_{j}}{SST}\times 100$$

This metric provides a direct measure of the proportion of the total variability explained by each factor.

The ANOVA analysis was performed independently for each response variable, allowing the identification of the most influential process parameters under different mechanical and thermomechanical conditions. The results derived from this analysis are presented and discussed in Section 3.

#### 2.4.2. Multi-Criteria Decision-Making (MCDM)

Given that multiple mechanical and thermomechanical performance indicators must be jointly evaluated to assess the experimental configurations, an MCDM approach was adopted. This methodology enables the integration of different performance indicators into a unified framework for the evaluation and ranking of the experimental configurations.

In MCDM problems, the determination of criteria weights plays a fundamental role in the final ranking of alternatives. These weights can be obtained using either subjective or objective approaches. Subjective methods, such as the Analytic Hierarchy Process (AHP) or Simple Multi Attribute Rating Technique (SMART) method, rely on expert judgment, which may introduce bias and reduce reproducibility. In contrast, objective methods, including the Entropy Weight Method (EWM) [53] or standard deviation-based approaches, determine the weights directly from the data, avoiding the influence of subjective assumptions.

In this study, the EWM was selected due to its ability to objectively quantify the relative importance of each criterion based on the intrinsic variability of the dataset. The fundamental principle of EWM is that criteria with greater dispersion or contrast among alternatives contain more information and, therefore, should be assigned higher weights. EWM assigns higher weights to criteria with higher information content (lower entropy), reducing bias in multi-objective optimization.

The procedure involves different steps. First, the decision matrix D = [x*_ij_*] is defined, where i=1,2, ...,n represents the experimental alternatives and i=j=1, 2, ...,m the evaluation criteria or response variables. Secondly, the matrix is then normalized in order to eliminate the influence of different scales by the normalized value of the *j*-th criterion for the *i*-th alternative, represented by Equation (6):

(6) $$p_{ij}=\frac{x_{ij}}{\sum_{i=1}^{n}x_{ij}}$$

Then, the entropy value of each criterion is calculated as per Equation (7):

(7) $$E_{j}=-k\sum_{i=1}^{n}p_{ij}\ln\left(p_{ij}\right)$$

where $k=\frac{1}{\ln\left(n\right)}$ is a constant that ensures the entropy values are bounded between 0 and 1. After that, the degree of diversification of each criterion is obtained as $1-E_{j}$, which reflects the amount of useful information contained in each variable.

Finally, the weight of each criterion is determined as Equation (8):

(8) $$w_{j}=\frac{1-E_{j}}{\sum_{j=1}^{m}\left(1-E_{j}\right)}$$

In this study, the Entropy Weight Method was applied to determine the objective weights of the selected response variables for both Z-PEEK and TPU 95A, based on the dispersion of the experimental data.

The weights obtained through the EWM are subsequently integrated into a MCDM framework in order to rank the experimental alternatives and identify the optimal set of process parameters. In this context, several well-established MCDM methods can be employed, such as Technique for Order Preference by Similarity to Ideal Solution (TOPSIS), Additive Ratio Assessment (ARAS) [54], and VIKOR.

TOPSIS is based on the concept of selecting the alternative that is closest to the ideal solution and farthest from the negative ideal solution. ARAS, on the other hand, evaluates alternatives based on their relative utility degree with respect to an optimal reference alternative.

TOPSIS is based on the selection of the alternative with the highest relative closeness to the ideal solution and the greatest distance from the negative ideal solution. ARAS, on the other hand, evaluates alternatives according to their relative utility degree with respect to an optimal reference alternative. These methods are widely used and effective for global performance evaluation. However, in the present study, the optimization problem involves criteria that may lead to competing requirements. For example, a parameter combination that improves maximum tensile force may not necessarily provide the lowest deformation or the highest stiffness retention at elevated temperature.

For this reason, VIKOR was selected as the primary MCDM method. VIKOR was specifically developed for multi-criteria problems involving conflicting criteria and compromise-solution analysis. Unlike approaches focused mainly on global proximity to an ideal solution, VIKOR introduces a compromise-ranking procedure that simultaneously considers the overall performance of each alternative, expressed as group utility, and the maximum individual deviation from the ideal solution, expressed as individual regret. This distinction is relevant for FFF parameter optimization because the selected printing condition should provide a balanced response among mechanical strength, deformation control and thermomechanical stiffness, while avoiding poor performance in any single criterion.

In addition, VIKOR includes validation conditions such as acceptable advantage and acceptable stability before a compromise solution is accepted. This feature provides an additional decision-control step compared with methods based only on relative closeness to the ideal solution. The suitability of VIKOR for this type of problem is also supported by previous applications in additive manufacturing and manufacturing process optimization. Raykar and D’Addona [55] and Deomore and Raykar [56] applied VIKOR-based decision-making to FDM printing parameter selection. Furthermore, Fernández et al. [57] compared several MCDM approaches, including ARAS, TOPSIS, VIKOR and COPRAS, for manufacturing parameter selection. Based on these considerations, VIKOR was considered suitable for identifying a balanced and interpretable optimal configuration under the multi-objective conditions evaluated in this work.

Firstly, the decision matrix $D=[x_{ij}]$ is defined following the same notation introduced in the entropy weighting method, where i=1,2, ...,n represents the experimental alternatives obtained from the DoE, and j=1,2, ...,m represents the evaluation criteria. In this study, the criteria correspond to maximum tensile force, maximum deformation, and storage modulus at 25 °C and 75 °C, while the weights $w_{j}$ are those previously obtained using the EWM.

Secondly, each criterion is classified according to its optimization objective as beneficial or cost criteria in order to reflect the desired performance objectives of the study. After that, the best $f_{j}^{*}$ and worst $f_{j}^{-}$ values for each criterion are determined as:

For benefit criterion, Equation (9) is used:

(9) $$f_{j}^{*}=\underset{i}{\max}\left(x_{ij}\right),{\;f}_{j}^{-}=\underset{i}{\min}\left(x_{ij}\right)$$

For cost criterion, Equation (10) is used:

(10) $$f_{j}^{*}=\underset{i}{\min}\left(x_{ij}\right),f_{j}^{-}=\underset{i}{\max}\left(x_{ij}\right)$$

where $f_{j}^{*}$ represents the ideal value and $f_{j}^{-}$ the anti-ideal value for each criterion.

Once the best $f_{j}^{*}$ and worst $f_{j}^{-}$ values for each criterion have been identified, the performance of each alternative is evaluated through the computation of the utility measure $S_{i}$ and the regret measure $R_{i}$.

The utility measure $S_{i}$ represents the overall deviation of each alternative from the ideal solution, considering all criteria simultaneously. It is calculated as Equation (11):

(11) $$S_{i}=\sum_{j=1}^{m}w_{j}\frac{f_{j}^{*}-x_{ij}}{f_{j}^{*}-f_{j}^{-}}$$

where $w_{j}$ denotes the weight of the j-th criterion obtained from the EWM. This formulation provides a global assessment of performance, where lower values of $S_{i}$ indicate solutions closer to the ideal condition across all criteria.

In contrast, the regret measure $R_{i}$ reflects the maximum individual deviation from the ideal solution among all criteria for each alternative, and is defined in Equation (12):

(12) $$R_{i}=\underset{j}{\max}\left[w_{j}\frac{f_{j}^{*}-x_{ij}}{f_{j}^{*}-f_{j}^{-}}\right]$$

This parameter captures the worst-case scenario, identifying the criterion for which each alternative performs the least favorably. Therefore, while $S_{i}$ evaluates the overall performance, $R_{i}$ emphasizes the most critical weakness of each solution.

This compromise logic is particularly relevant in FFF parameter optimization because the selected processing conditions may affect the response variables in different and sometimes competing ways. For example, a parameter combination that improves maximum tensile force may not necessarily provide the lowest deformation or the highest stiffness retention at elevated temperature. Therefore, the optimal alternative should not be interpreted as the experiment that maximizes a single response, but as the configuration that provides the most balanced performance across all selected criteria. In this sense, the utility measure $S_{i}$ accounts for the global distance from the ideal solution, whereas the regret measure $R_{i}$ prevents the selection of alternatives with a strong weakness in one specific criterion. The final VIKOR index $Q_{i}$ therefore identifies a compromise solution that balances mechanical strength, deformation control and thermomechanical stiffness, with an integration of both indicators into a single compromise metric as shown in Equation (13).

The VIKOR index is defined as:

(13) $$Q_{i}=v\frac{S_{i}-S^{*}}{S^{-}-S^{*}}+\left(1-v\right)\frac{R_{i}-R^{*}}{R^{-}-R^{*}}$$

where $S^{*}=min\;S_{i}$ and $S^{-}=max\;S_{i}$ represent the best and worst values of the utility measure, respectively, while $R^{*}=min\;R_{i}$ and $R^{-}=max\;R_{i}$ correspond to the best and worst values of the regret measure. In this study, the VIKOR parameter v was set to 0.5. This value represents a balanced compromise between the group utility measure $S_{i}$ and the individual regret measure $R_{i}$, without prioritizing either the majority criterion or the worst-performing criterion. This choice is consistent with the objective of identifying a compromise solution among mechanical strength, deformation control and thermomechanical stiffness. Once the VIKOR index $Q_{i}$ is computed, the experimental alternatives are ranked accordingly to their proximity to the ideal solution, where lower values of $Q_{i}$ indicate better overall performance. In this work, it enables the identification of the optimal printing configuration by simultaneously considering strength, deformability, and temperature-dependent stiffness. Therefore, the ranking obtained from $Q_{i}$ directly reflects the best compromise between mechanical and thermomechanical performance.

To ensure the robustness of the selected solution, the VIKOR method introduces two additional validation conditions. The first one, known as the acceptable advantage condition, evaluates whether the best-ranked alternative exhibits a sufficient improvement over the second-ranked one, and is defined in Equation (14):

(14) $$Q\left(a_{2}\right)-Q\left(a_{1}\right)\geq \frac{1}{n-1}$$

where $a_{1}$ and $a_{2}$ are the first and second alternatives in the ranking, and n is the total number of alternatives.

The second condition, referred to as the acceptable stability condition, requires that the best alternative according to $Q_{i}$ is also ranked favorably in terms of either the utility measure $S_{i}$ or the regret measure $R_{i}$. This condition ensures that the selected solution is not only optimal in a combined sense but also stable with respect to individual performance indicators.

Only when both conditions are satisfied can the identified alternative be considered the optimal compromise solution. Otherwise, a set of near-optimal solutions may be proposed.

## 3. Results and Discussion

This section presents and discusses the results obtained from the experimental and analytical framework described in Section 2. The analysis is structured to progressively evaluate the influence of the FFF process parameters on the mechanical and thermomechanical behavior of the investigated materials, followed by the identification of optimal processing conditions.

### 3.1. Experimental Mechanical and Thermomechanical Results

Firstly, the experimental results from the tensile and DMA tests for Z-PEEK and TPU 95A are shown in Table 6 and Table 7, respectively. The values reported in Table 6 and Table 7 correspond to the mean ± standard deviation obtained from three independent repetitions for each experimental condition. The specimens associated with each experimental condition were printed within the same printing batch in order to minimize process-related variability. The inclusion of the standard deviation allows the experimental dispersion to be evaluated and provides a more robust basis for interpreting the subsequent ANOVA results. This is particularly relevant for those factors showing *p*-values close to the significance threshold, for which the statistical relevance must be interpreted together with the observed experimental variability.

The analysis of both materials reveals significantly different behaviors, as expected from their distinct microstructural and viscoelastic characteristics.

In the case of Z-PEEK, the material exhibits high stiffness and relatively low deformation, with an average maximum tensile force of 592.90 N and a maximum deformation of 3.42 mm. These values are consistent with the behavior of a semicrystalline high-performance thermoplastic, where load-bearing capacity is dominant over ductility. From a thermomechanical perspective, the storage modulus shows a clear decreasing trend with increasing temperature, from 3433.54 MPa at 25 °C to 223.29 MPa at 200 °C, which represents an average loss of 93.5%, reflecting the progressive reduction in stiffness as the material approaches and exceeds its glass transition region. The tan δ values, with an average of 0.26 at 174 °C, indicate a moderate viscoelastic response, with a non-negligible contribution of energy dissipation mechanisms and a limited damping capacity within the evaluated temperature range. Additionally, the relatively low dispersion of the results suggests good process repeatability and consistent interlayer adhesion.

In contrast, TPU 95A exhibits a markedly different behavior, characterized by lower stiffness and significantly higher deformability, with an average maximum tensile force of 221.37 N and a maximum deformation of 156.37 mm. This behavior is typical of elastomeric materials, where flexibility and energy absorption capabilities dominate over strength. The thermomechanical analysis further highlights this difference, as the storage modulus decreases from 140.72 MPa at 25 °C to 11.12 MPa at 150 °C, indicating a pronounced sensitivity to temperature. The tan δ values for TPU, with an average of 0.19 ± 0.04, together with the associated temperature of the tan δ peak (162.04 °C), reflect a predominantly elastic response with a limited but non-negligible viscoelastic contribution under the evaluated conditions. Although TPU is intrinsically elastomeric, the experimental results show that, at the evaluated temperature, Z-PEEK exhibits a higher tan δ value (≈0.26 ± 0.08) compared to TPU (≈0.19 ± 0.04), indicating a relatively greater contribution of energy dissipation mechanisms, likely associated with its proximity to the glass transition region.

Overall, the results indicate that both materials exhibit a clear dependence on temperature and processing conditions, although with markedly different behaviors. These observations highlight the importance of considering both mechanical and thermomechanical indicators when evaluating FFF-manufactured components. A more detailed assessment of the influence of the printing parameters on these responses is carried out through statistical analysis.

### 3.2. Statistical Analysis (ANOVA)

To quantitatively assess the influence of the selected printing parameters on the mechanical and thermomechanical responses, an ANOVA was performed for both materials. This approach allows the identification of statistically significant factors and the quantification of their relative contribution to the variability of each response variable, providing a quantitative understanding of the process–property relationships. A confidence level of 95% (*p* < 0.05) was considered to determine statistical significance.

The analyses were conducted independently for Z-PEEK and TPU 95A, taking into account their respective DoE configurations. The evaluated response variables include tensile properties (maximum force and deformation) and thermomechanical indicators (tan δ, tan δ temperature, and storage modulus at different temperatures).

#### 3.2.1. TPU 95A ANOVA Discussion

For TPU 95A, the ANOVA results, shown in Table 8, reveal that the influence of the printing parameters varies significantly depending on the response variable.

In the case of maximum tensile force, the extrusion temperature was identified as the only statistically significant factor (*p* = 0.0263), with a contribution of approximately 27.05%, indicating a dominant role in determining the load-bearing capacity of the material. The remaining parameters, including printing speed, build plate temperature, and layer height, showed lower contributions and were not statistically significant.

For the maximum deformation, the layer height was found to be the most influential parameter (*p* = 0.0442, contribution ≈ 24.8%), highlighting its relevance in controlling the ductility and flexibility of TPU structures. Other parameters exhibited minor contributions and were not statistically significant.

Regarding the thermomechanical behavior, the analysis of tan δ indicates that none of the evaluated parameters have a statistically significant effect, with very low contributions across all factors. This suggests that the damping behavior of TPU is primarily governed by its intrinsic material properties rather than processing conditions.

Finally, the analysis of the storage modulus at different temperatures shows a temperature-dependent influence of the parameters. In particular, the extrusion temperature becomes statistically significant at elevated temperatures, such as 100 °C (*p* = 0.0254) and 150 °C (*p* = 0.042), with contributions exceeding 23% and 27%, respectively. This indicates that processing temperature plays a key role in defining the thermal stability and stiffness evolution of TPU at higher temperatures.

It should also be noted that, although several TPU 95A parameters were not statistically significant for some response variables, this result has practical relevance from a process-optimization perspective. Within the investigated parameter ranges, high *p*-values indicate that moderate variations in these factors produced limited changes in the corresponding mechanical or thermomechanical response. Therefore, these factors may define processing regions with low performance sensitivity, where small deviations in printing conditions can be tolerated without substantially affecting part performance.

From a manufacturing perspective, such regions are advantageous because they provide a wider and more flexible processing window rather than a narrowly defined optimum. This behavior improves process robustness and operational flexibility, particularly in elastomeric FFF materials such as TPU 95A, where stable feeding, filament continuity and interlayer cohesion are essential for repeatable manufacturing. Consequently, the non-significant factors identified in Table 8 should also be interpreted as indicators of parameter tolerance within the evaluated experimental domain.

#### 3.2.2. Z-PEEK ANOVA Discussion

For Z-PEEK, the ANOVA results indicate a different behavior, with a stronger dependence on thermal processing conditions, particularly those related to the thermal environment during printing. These results are shown in Table 9.

In the case of Z-PEEK, the ANOVA results reveal that the influence of the printing parameters is strongly governed by the thermal history of the material during processing, particularly under the specific conditions of thin-walled specimens fabricated with a support interface between the build plate and the part.

For the tensile behavior, the build plate temperature emerges as the most influential parameter, showing the highest contribution (≈27%). This can be explained by its direct role in controlling the initial cooling rate and interlayer bonding conditions. In thin geometries, heat dissipation is significantly faster due to the reduced thermal mass, making the first layers especially sensitive to the thermal boundary conditions imposed by the build plate. A higher plate temperature promotes improved adhesion and reduces thermal gradients, leading to enhanced load transfer across layers.

This effect is further amplified by the presence of a support structure between the plate and the specimen, which introduces an additional thermal interface. The support material modifies the heat transfer path, acting as a partial thermal barrier and altering the effective cooling conditions experienced by the printed part. As a result, the combined effect of plate temperature and support geometry becomes critical in defining the final mechanical performance.

In contrast, for the thermomechanical properties, particularly the storage modulus at elevated temperatures, the chamber temperature becomes the dominant factor. This behavior is consistent with the processing characteristics of semicrystalline polymers such as PEEK, where the degree of crystallinity is highly dependent on the thermal environment during solidification. A higher chamber temperature reduces thermal gradients and promotes more uniform crystallization, which directly influences the stiffness retention at elevated temperatures.

This effect becomes more pronounced at higher testing temperatures (150–200 °C), where the material approaches its glass transition region and the influence of the crystalline phase becomes more critical. Under these conditions, the chamber temperature governs the thermal relaxation and crystallization kinetics, explaining its dominant contribution in the ANOVA results.

Regarding the viscoelastic response (tan δ), the relatively low contribution of all parameters suggests that this property is primarily governed by the intrinsic molecular structure of the material, rather than by processing conditions. This is consistent with the behavior of semicrystalline polymers, where damping characteristics are less sensitive to moderate variations in processing parameters.

Overall, the observed trends indicate that, for thin-walled FFF-printed PEEK components, the interaction between thermal boundary conditions (build plate), global thermal environment (chamber), and heat transfer through support structures plays a critical role in defining both mechanical and thermomechanical performance.

Finally, the ANOVA results demonstrate that the influence of the printing parameters is highly dependent on both the material type and the specific response variable. While certain parameters exhibit statistically significant effects under specific conditions, the interaction between mechanical and thermomechanical responses highlights the need for a multi-objective optimization approach. Therefore, an MCDM methodology is applied in the following section to determine the optimal set of printing parameters.

### 3.3. Multi-Criteria Decision-Making

To ensure an objective and data-driven analysis, the weighting of the response variables was determined using the EWM, as it assigns higher importance to criteria with greater variability, under the assumption that such variables provide more discriminative information for the decision-making process.

Subsequently, the VIKOR method was applied to identify the optimal compromise solution, considering both group utility and individual regret measures.

#### 3.3.1. Entropy Weight Method (EWM)

For both materials, a reduced set of four response variables was considered in the MCDM analysis, namely maximum tensile force (F_max_), maximum deformation (Def_max_) and storage modulus at 25 °C and 75 °C. This selection was made according to the application-oriented scope of the study. The selected criteria were intended to represent load-bearing capacity, deformation response, room-temperature stiffness and stiffness retention under moderately elevated service-temperature conditions. This reduced set of criteria allows the optimization framework to account for both mechanical performance and thermomechanical stability without including the complete DMA temperature range in the decision matrix.

Storage modulus values measured at different temperatures are related because they originate from the same DMA curve. Therefore, E′ at 25 °C and E′ at 75 °C should not be interpreted as statistically independent variables in a strict correlation sense. However, in the present MCDM framework, they were retained as independent decision criteria because they represent different engineering requirements. E′ at 25 °C describes the initial stiffness of the printed specimens under room-temperature conditions, whereas E′ at 75 °C describes stiffness retention under moderately elevated temperature conditions.

Although E′ generally decreases with increasing temperature, the reduction from 25 °C to 75 °C does not follow the same proportionality for all printing conditions. The magnitude of this decrease depends on the selected FFF parameters and differs between Z-PEEK and TPU 95A. Consequently, E′ at 75 °C provides information about the ability of each printed condition to preserve stiffness with temperature, which is not fully captured by E′ at 25 °C alone.

To minimize the risk of overrepresenting a single thermomechanical dimension, only two storage modulus indicators were included in the main EWM–VIKOR ranking. The higher-temperature E′ values measured by DMA were retained for thermomechanical characterization and discussion, but were not included as primary optimization criteria. Therefore, potential correlation between E′ at 25 °C and E′ at 75 °C is acknowledged, but it is not expected to materially dominate the final ranking because the decision matrix remains limited to two physically interpretable stiffness states together with the mechanical criteria F_max_ and Def_max_.

The optimization direction of each criterion was defined according to this application-oriented framework. F_max_ and storage modulus values were treated as benefit criteria, since higher load-bearing capacity and higher stiffness retention are desirable. In contrast, Def_max_ was treated as a cost criterion for both Z-PEEK and TPU 95A, meaning that lower maximum deformation values were preferred. This decision was based on the requirements of functional components operating under mechanical loading, where excessive deformation may compromise dimensional stability, geometric accuracy, load transmission and long-term performance. Therefore, the optimization objective was not to maximize deformation capacity, but to identify printing conditions providing a controlled deformation response together with adequate strength and thermomechanical stiffness.

The loss factor tan δ was also excluded from the main VIKOR ranking because its optimization direction is application-dependent. Higher tan δ values may be desirable in damping-oriented applications, whereas lower values may be preferred when elastic recovery, reduced hysteresis or dimensional stability are prioritized. Consequently, tan δ was used as a descriptive thermomechanical parameter in the discussion of the DMA results, but it was not considered as a direct benefit or cost criterion in the main MCDM optimization.

The EWM was then applied to the experimental dataset following the formulation described in Equations (1)–(5). Based on the normalized decision matrix, the entropy values, diversification degrees, and corresponding weights were calculated for each response variable. The resulting weights reflect the relative importance of each criterion, as determined exclusively by the intrinsic variability of the data. In this context, criteria exhibiting greater dispersion across the experimental conditions are assigned to higher weights, as they provide a stronger discriminatory capability between alternatives.

Table 10 summarizes the entropy values and final weights obtained for each response variable. The normalized decision matrix is not included for brevity, as the analysis focuses on the resulting weights, which directly govern the subsequent MCDM evaluation.

The entropy weighting results reveal a markedly different behavior between the two materials. In the case of Z-PEEK, the most influential criteria correspond to the storage modulus at 25 °C and 75 °C, with weights of 33.26% and 34.10%, respectively. In contrast, the mechanical parameters—maximum tensile force and deformation—present significantly lower contributions, with values of 13.04% and 19.60%, respectively.

This distribution reflects the intrinsic characteristics of semicrystalline high-performance polymers, where the mechanical response is strongly governed by the degree of crystallinity and thermal history during processing. Small variations in processing conditions can lead to significant differences in crystalline structure, which directly affect the stiffness of the material, particularly at elevated temperatures. As a result, the thermomechanical indicators exhibit greater dispersion across the experimental dataset, and consequently, higher entropy weights. Conversely, the tensile strength shows comparatively lower variability, suggesting a more stable behavior with respect to the evaluated printing parameters.

In contrast, TPU 95A exhibits a fundamentally different trend. The maximum tensile force emerges as the dominant criterion, with a weight of 41.29%, while the remaining variables—deformation and storage modulus at both temperatures—show more balanced contributions, all around 20%.

This behavior is consistent with the nature of elastomeric polymers, where the mechanical response is primarily controlled by chain mobility and large deformation mechanisms rather than stiffness. In this case, the higher variability in tensile force indicates a strong sensitivity to processing parameters, particularly in terms of interlayer bonding and filament fusion quality, which directly affect the load-bearing capacity of the material. On the other hand, the relatively uniform contribution of the remaining parameters suggests that stiffness-related properties are less sensitive to the evaluated processing conditions, being more strongly governed by the inherent viscoelastic nature of the material.

Overall, these results highlight a key finding: while Z-PEEK performance is dominated by thermomechanical behavior, particularly stiffness retention with temperature, TPU 95A is primarily governed by mechanical performance under large deformation conditions. This contrast demonstrates that the relative importance of performance criteria is strongly material-dependent, reinforcing the need for tailored optimization strategies in FFF processes depending on the polymer type.

#### 3.3.2. VIKOR

Following the determination of objective weights through the EWM, the VIKOR method was applied to rank the experimental alternatives and identify the optimal set of process parameters. The analysis was performed according to the formulation described in Equations (6)–(13), using the same decision matrix and criteria defined previously.

The VIKOR approach enables the identification of a compromise solution by simultaneously considering the overall performance of each alternative and the maximum individual deviation among criteria. This is particularly relevant in the present study, where multiple and potentially conflicting performance indicators—such as mechanical strength, deformability, and temperature-dependent stiffness—must be optimized simultaneously.

Based on the calculated utility measure (S_i_), regret measure (R_i_), and VIKOR index (Q_i_), the experimental configurations were ranked for each material. Table 11 and Table 12 summarize the obtained values and the corresponding ranking of alternatives.

The optimal solution was identified according to the acceptable advantage and acceptable stability conditions, ensuring the robustness of the selected parameter combination.

For Z-PEEK, the best-ranked alternative corresponds to Experiment 17 (ET = 400 °C, PT = 200 °C, and CT = 150 °C), which presents the lowest $Q_{i}$ value. However, this result should not be interpreted only as the numerical minimum of the VIKOR index, but as a compromise solution that balances the global utility $S_{i}$ and the individual regret $R_{i}$. In this case, Experiment 17 combines high maximum tensile force, controlled deformation and high storage modulus values at both 25 °C and 75 °C. Therefore, the selected configuration provides a balanced response across the selected criteria rather than maximizing a single isolated property.

This finding is consistent with the entropy weighting results, where the storage modulus at 25 °C and 75 °C was identified as the dominant criterion.

It is also in strong agreement with the ANOVA results, which revealed that thermally driven parameters—particularly the chamber temperature—have a dominant influence on the variability of the thermomechanical response. In this context, the optimal chamber temperature of 150 °C is especially relevant, as it is very close to the glass transition temperature of Z-PEEK (≈143 °C). Operating in this temperature range promotes increased molecular mobility during deposition, which enhances reptation and interdiffusion across filament interfaces, facilitating interlayer diffusion and stress relaxation, while still enabling controlled crystallization during cooling.

**Figure 3:**
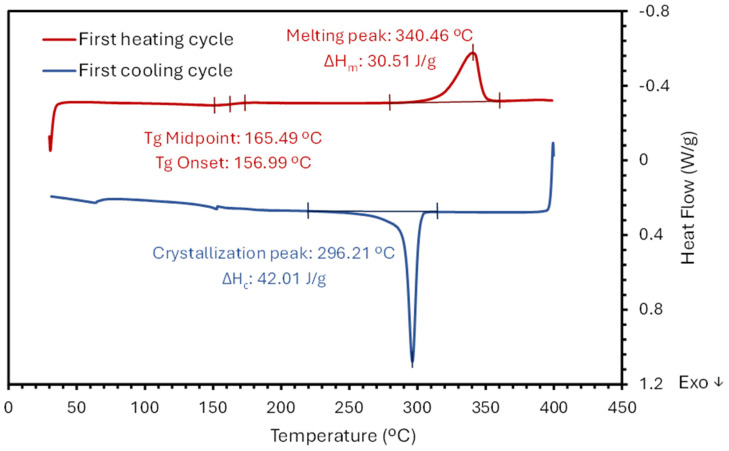
DSC thermogram of the optimized Z-PEEK specimen manufactured using ET = 400 °C, PT = 200 °C, and CT = 150 °C. The thermogram shows the characteristic glass transition region, melting peak, and crystallization behavior of the printed material.

To support the interpretation of the optimized Z-PEEK printing condition, an additional DSC analysis was performed on specimens manufactured using the optimal parameter combination identified by the MCDM analysis: ET = 400 °C, PT = 200 °C, and CT = 150 °C. The DSC thermogram is shown in Figure 3. The curve shows the characteristic thermal transitions of semicrystalline PEEK, including a glass transition region and a melting peak at approximately 340.48 °C.

The degree of crystallinity was estimated from the melting enthalpy obtained during the first heating cycle according to Equation (15):

(15) $$\;X_{C}\left(\%\right)=\frac{{\Delta H}_{m}-{\Delta H}_{cc}}{{\Delta H}_{m}^{0}}\;$$

where $\Delta H_{m}$ is the melting enthalpy obtained from the melting peak during the heating cycle, $\Delta H_{cc}$ is the cold crystallization enthalpy associated with crystallization phenomena occurring during heating, and $\Delta H_{m}^{0}$ is the theoretical melting enthalpy of fully crystalline PEEK, taken as 130 J/g according to Blundell and Osborn [58].

For the optimized Z-PEEK specimen, the melting enthalpy was 30.51 J/g. Since no significant cold crystallization peak was observed during the first heating cycle, $\Delta H_{cc}\;$was considered negligible. The resulting degree of crystallinity was therefore approximately 23.5%. This result confirms the presence of a semicrystalline structure in the optimized printed Z-PEEK specimen and supports the relevance of the thermal processing conditions in the final thermomechanical response.

Previous studies have shown that the thermal history imposed during FFF processing of PEEK significantly affects crystallinity and mechanical performance. In this context, Yang et al. [59] reported that favorable thermal processing conditions promote crystallinity development in FFF-processed PEEK, demonstrating the strong influence of printing thermal conditions on the final material response.

It should be noted that DSC provides information on thermal transitions and crystallinity, but does not directly characterize interlayer morphology, porosity, or interlayer bonding.

From a processing perspective, this configuration creates a favorable thermal environment that reduces temperature gradients between the extruded filament and the surrounding material. The high plate temperature (200 °C) further contributes to minimizing thermal gradients at the deposition interface, reducing residual stresses and enhancing interlayer bonding. At the same time, the intermediate extrusion temperature (400 °C), within the manufacturer-recommended range, ensures adequate melt viscosity, enabling sufficient molecular interdiffusion without compromising geometrical stability. These combined effects explain the improved thermomechanical stability observed in the optimized condition.

In contrast, for TPU 95A, the optimal solution corresponds to Experiment 2 (ET = 225 °C, LH = 0.1 mm, PS = 50 mm/s, and PT = 80 °C), characterized by the lowest $Q_{i}$ value. As in the case of Z-PEEK, this alternative should be interpreted as a compromise solution rather than as the isolated maximization of a single response variable. Experiment 2 provides the best balance between load-bearing capacity, controlled deformation and thermomechanical stiffness within the evaluated TPU 95A design space.

This result is in agreement with the entropy analysis, where maximum tensile force was identified as the most influential criterion. Therefore, the VIKOR ranking indicates that, for TPU 95A, the optimal compromise is mainly driven by mechanical performance, while still maintaining an acceptable deformation response and stiffness retention at the selected temperatures.

The VIKOR results are also consistent with the ANOVA analysis, which indicated that process parameters associated with material deposition—such as extrusion temperature and printing speed—have a stronger influence on the mechanical response than thermally driven parameters. This behavior is characteristic of elastomeric materials, where mechanical performance is primarily governed by filament continuity and interlayer cohesion rather than stiffness retention.

Unlike Z-PEEK, TPU 95A optimization is not driven by thermal transitions, but by the ability to achieve stable and uniform material deposition. In this sense, the selected combination of moderate extrusion temperature, low layer height, and intermediate printing speed enhances filament control and interlayer contact, leading to improved load-bearing capacity. Higher extrusion temperatures may lead to excessive filament softening, reducing deposition stability and geometric accuracy, which negatively affects interlayer cohesion. Moreover, operating close to the upper thermal limit of TPU (with degradation onset typically reported around 260 °C) may induce early-stage thermal degradation mechanisms, further compromising interfacial bonding and mechanical performance.

In addition, the robustness of the obtained optimal solutions was verified through the VIKOR validation conditions. For both materials, the acceptable advantage condition was satisfied, indicating that the difference between the best-ranked and the second-best alternatives is sufficiently significant to ensure a clear ranking.

Similarly, the acceptable stability condition was also fulfilled, as the top-ranked alternatives are consistent with the rankings obtained from the utility $S_{i}$ and/or regret $R_{i}$ measures. This confirms that the identified optimal configurations represent stable compromise solutions, reinforcing the reliability of the VIKOR-based decision-making process.

As an additional robustness check, the VIKOR ranking was recalculated using the standard deviation weighting method. This calculation was not intended to establish a comparative assessment of weighting methods, but to evaluate whether the selected optimal configurations were sensitive to the entropy-based weighting scheme. The standard deviation method produced a more balanced distribution of weights, with values close to 0.25 for the four selected criteria. Despite this modification, the same optimal alternatives were obtained for both materials: Experiment 17 for Z-PEEK and Experiment 2 for TPU 95A. This result indicates that the selected optimal parameters are not exclusively driven by the EWM weighting scheme and supports the robustness of the MCDM conclusions within the scope of the present study.

These results confirm that the optimal printing strategy is strongly material-dependent, with thermomechanical performance dominating in semicrystalline polymers such as PEEK, and mechanical response under large deformation governing elastomeric materials such as TPU.

## 4. Conclusions and Future Works

This work developed and validated a comprehensive experimental–statistical framework for the optimization of FFF process parameters, integrating Design of Experiments (DoE), mechanical and thermomechanical characterization, Analysis of Variance (ANOVA), the Entropy Weight Method (EWM), and the VIKOR method into a unified Multi-Criteria Decision-Making approach. The main methodological contribution lies in incorporating DMA-derived temperature-dependent stiffness indicators into the decision matrix, allowing the optimization to move beyond room-temperature mechanical performance and toward service-oriented parameter selection. This integration enables the identification of compromise solutions that balance load-bearing capacity, deformation response and stiffness retention under relevant thermal conditions.

The results demonstrate that the relative importance of performance indicators is strongly material-dependent. For Z-PEEK, the entropy-based weighting revealed that the dominant criteria are the storage modulus values at 25 °C and 75 °C, indicating that stiffness retention under thermal loading governs the optimization process. This finding is consistent with the ANOVA results, which identified thermally driven parameters—particularly the chamber temperature—as the most influential factors affecting the thermomechanical response.

The optimal processing conditions for Z-PEEK (400 °C extrusion temperature, 200 °C plate temperature, and 150 °C chamber temperature) can be directly explained by the thermal behavior of semicrystalline polymers. The intermediate extrusion temperature within the manufacturer-recommended range ensures adequate melt viscosity and molecular interdiffusion without inducing excessive thermal degradation. The elevated plate temperature reduces thermal gradients at the deposition interface, minimizing residual stresses and promoting interlayer adhesion. Most importantly, the chamber temperature close to the glass transition temperature of Z-PEEK (≈143 °C) provides a favorable thermal environment for stress relaxation and controlled crystallization, leading to enhanced thermomechanical stability.

In contrast, for TPU 95A, the entropy analysis identified the maximum tensile force as the dominant criterion, highlighting that the optimization of elastomeric systems is primarily governed by load-bearing capacity rather than stiffness retention. This is supported by the ANOVA results, where process parameters related to material deposition —such as extrusion temperature and printing speed—exhibited the highest influence on the mechanical response.

The optimal TPU configuration (225 °C extrusion temperature, 0.1 mm layer height, 50 mm/s printing speed, and 80 °C plate temperature) reflects a balance between material flow and deposition stability. Moderate extrusion temperatures enhance filament continuity and interlayer bonding, while avoiding excessive softening. Higher temperatures, although within the nominal processing range, approach the onset of thermal degradation (≈260 °C), which may negatively affect material integrity. Additionally, reduced layer height improves interlayer contact and bonding quality, while intermediate printing speeds ensure stable material deposition. These factors collectively maximize tensile load capacity under controlled deformation conditions.

Overall, the results confirm that optimal FFF processing strategies cannot be generalized across materials without considering their intrinsic thermomechanical behavior. In semicrystalline high-performance polymers such as PEEK, optimization is dominated by thermal history, crystallization kinetics, and stiffness retention. In contrast, elastomeric materials such as TPU are governed by deposition stability, filament continuity and interlayer cohesion.

It is important to distinguish between the specific parameter recommendations obtained in this study and the broader methodological contribution of the proposed framework. Under these controlled conditions, the selected configurations represent recommended parameter sets for the specific Z-PEEK/Zortrax Endureal and TPU 95A/Ultimaker S5 combinations investigated in this work. If different printers, material suppliers, filament batches or processing environments are used, additional validation or adjustment of the numerical parameter values may be required. Nevertheless, the proposed DoE–ANOVA–EWM–VIKOR methodology remains directly transferable, since the same workflow can be applied to other FFF systems by redefining the corresponding factors, levels and feasible operating windows.

Future work will focus on extending the proposed framework through its integration with numerical simulation tools, enabling the predictive design of optimized FFF components. In this context, the experimental results obtained in this study will serve as a foundation for the development and validation of real industrial prototypes, particularly in applications requiring tailored thermomechanical performance.

More specifically, the optimized printing conditions identified in this work will be applied to the fabrication of a flexible coupling intended to operate in contact with refrigerants at temperatures above room temperature, with an expected service range around 75 °C. This target application provides the engineering basis for selecting E′ at 75 °C as the representative elevated-temperature stiffness criterion in the MCDM framework. Future studies will therefore focus on validating the optimized configurations under service-like conditions, including exposure to relevant working media, cyclic mechanical loading, thermal aging and long-term dimensional and mechanical stability.

Although the present MCDM framework was intentionally focused on mechanical and thermomechanical response variables, future extensions should incorporate manufacturing-related criteria such as production time, energy consumption, dimensional accuracy, process stability, material consumption, and production cost. The inclusion of these indicators will allow the optimization procedure to evolve from a thermomechanical performance-oriented framework toward a more comprehensive industrial decision-making tool for the final design and validation of the flexible coupling.

This approach aims to bridge the gap between experimental optimization and practical implementation, contributing to the deployment of advanced additively manufactured components in industrial environments.

## Figures and Tables

**Table 1:** Z-PEEK and TPU 95A properties.

Property	Unit	Z-PEEK	TPU 95A
Density	g/cm^3^	1.30	1.22
Tensile Modulus	MPa	3720	67
Elongation at Break	%	28	>560
Flexural Strength	MPa	130	4.1
Flexural Modulus	MPa	2700	55.1
Glass Transition Temperature (T_g_)	°C	143	-
Heat Deflection Temperature (HDT)	°C	160 (at 1.80 MPa)	50.3 (at 0.455 MPa)
Melting Temperature (T_m_)	°C	343	216.8

**Table 2:** FFF parameters for Z-PEEK 3D design levels.

Variable	Notation	Unit	Level		
			−1	0	1
Extrusion Temperature	ET	°C	375	400	425
Chamber Temperature	CT	°C	125	150	170
Plate Temperature	PT	°C	140	170	200

**Table 3:** Printing parameters used for Z-PEEK DoE.

Experiment No.	Input Variables for Z-PEEK Experiments			Suitability *
	ET [°C]	PT [°C]	CT [°C]	
1	375	140	125	YES
2	375	140	150	NO
3	375	140	170	NO
4	375	170	125	YES
5	375	170	150	YES
6	375	170	170	YES
7	375	200	125	YES
8	375	200	150	YES
9	375	200	170	YES
10	400	140	125	YES
11	400	140	150	NO
12	400	140	170	NO
13	400	170	125	YES
14	400	170	150	YES
15	400	170	170	YES
16	400	200	125	YES
17	400	200	150	YES
18	400	200	170	YES
19	425	140	125	YES
20	425	140	150	NO
21	425	140	170	NO
22	425	170	125	YES
23	425	170	150	YES
24	425	170	170	YES
25	425	200	125	YES
26	425	200	150	YES
27	425	200	170	YES

Note (*): Suitability indicates whether the experiment can be performed (‘Yes’) or not (‘No’). In all infeasible cases, CT > PT (Chamber Temperature > Plate Temperature), which is not possible due to the chamber heating mechanism through the build plate.

**Table 4:** FFF parameters for TPU 95A 3D design levels.

Variable	Notation	Unit	Level		
			−1	0	1
Extrusion Temperature	ET	°C	225	240	255
Layer Height	LH	mm	0.1	0.15	0.2
Printing Speed	PS	mm/s	20	50	80
Plate Temperature	PT	°C	60	70	80

**Table 5:** DoE for TPU 95A samples.

Experiment No.	Input Variables for TPU 95A Experiments			
	ET [°C]	LH [mm]	PS [mm/s]	PT [°C]
1	225	0.10	20	70
2	225	0.10	50	80
3	225	0.10	80	60
4	225	0.15	20	80
5	225	0.15	50	60
6	225	0.15	80	70
7	225	0.20	20	60
8	225	0.20	50	70
9	225	0.20	80	80
10	240	0.10	20	80
11	240	0.10	50	60
12	240	0.10	80	70
13	240	0.15	20	60
14	240	0.15	50	70
15	240	0.15	80	80
16	240	0.20	20	70
17	240	0.20	50	80
18	240	0.20	80	60
19	255	0.10	20	60
20	255	0.10	50	70
21	255	0.10	80	80
22	255	0.15	20	70
23	255	0.15	50	80
24	255	0.15	80	60
25	255	0.20	20	80
26	255	0.20	50	60
27	255	0.20	80	70

**Table 6:** Mechanical and thermomechanical characterization results for Z-PEEK specimens. Values are reported as mean ± standard deviation from three independent repetitions.

Experiment No.	Output Variables for Z-PEEK							
	F_max_ (N)	Def_max_ (mm)	E′_25°C_ [MPa]	E′_75°C_ [MPa]	E′_100°C_ [MPa]	E′_150°C_ [MPa]	E′_200°C_ [MPa]	Tan δ
1	559.06 ± 18.51	3.5136 ± 0.13	3947.72 ± 55.12	3724.66 ± 30.46	3596.60 ± 19.43	2268.02 ± 106.8	272.94 ± 6.08	0.299
2	---------------------------------------------------------------------------------------------------------------------------------------------------							
3	---------------------------------------------------------------------------------------------------------------------------------------------------							
4	573.94 ± 14.10	2.9967 ± 0.02	3203.77 ± 21.42	3024.61 ± 120.0	2911.22 ± 34.50	1796.44 ± 81.95	230.47 ± 2.50	0.227
5	543.17 ± 12.67	3.4012 ± 0.10	3481.75 ± 43.69	3278.33 ± 73.91	3165.52 ± 16.20	2375.00 ± 21.86	204.64 ± 5.19	0.418
6	522.74 ± 3.74	3.7417 ± 0.09	2813.59 ± 48.73	2656.11 ± 121.0	2583.23 ± 41.30	1120.00 ± 121.9	137.67 ± 8.95	0.345
7	644.06 ± 12.20	3.7781 ± 0.04	3317.20 ± 39.09	3169.42 ± 74.23	3074.53 ± 115.33	2185.75 ± 13.59	239.64 ± 5.56	0.196
8	641.27 ± 9.45	4.2931 ± 0.03	2952.83 ± 46.60	2793.72 ±107.68	2707.02 ± 59.30	2180.64 ± 117.2	320.62 ± 2.17	0.130
9	587.31 ± 14.05	3.5044 ± 0.11	3024.81 ± 133.03	2807.95 ± 46.67	2670.61 ± 45.57	1547.54 ± 55.00	144.29 ± 4.10	0.310
10	498.40 ± 11.42	3.7606 ± 0.15	2503.70 ± 83.43	2367.80 ± 105.8	2319.11 ± 84.32	1053.11 ± 38.31	100.86 ± 4.71	0.361
11	---------------------------------------------------------------------------------------------------------------------------------------------------							
12	---------------------------------------------------------------------------------------------------------------------------------------------------							
13	561.59 ± 2.33	2.7360 ± 0.07	4256.53 ± 135.85	4042.95 ± 138.2	3926.42 ± 117.07	2905.93 ± 154.1	277.60 ± 9.14	0.269
14	605.77 ± 0.28	3.4479 ± 0.10	4346.45 ± 133.03	4009.49 ± 166.5	3921.66 ± 123.42	2783.24 ± 42.08	253.54 ± 4.69	0.327
15	608.92 ± 13.93	3.2878 ± 0.05	3295.03 ± 154.41	2980.52 ± 92.18	2875.48 ± 106.30	1176.20 ± 53.85	156.08 ± 6.50	0.300
16	607.54 ± 19.33	3.2193 ± 0.05	3394.70 ± 84.71	3190.22 ± 37.70	3087.41 ± 104.61	1950.18 ± 118.3	265.58 ± 1.02	0.177
17	694.21 ± 20.16	3.3499 ± 0.12	4422.92 ± 209.16	4168.51 ± 50.41	4010.18 ± 49.63	3104.20 ± 121.6	247.35 ± 12.83	0.391
18	671.93 ± 24.49	3.4982 ± 0.03	3761.95 ± 179.92	3481.33 ± 42.04	3386.13 ± 101.94	2251.81 ± 72.13	257.02 ± 16.31	0.187
19	616.29 ± 4.32	3.8298 ± 0.12	3123.13 ± 28.19	2822.87 ±97.49	2738.19 ± 150.90	2185.43 ± 148.4	269.31 ± 12.75	0.172
20	---------------------------------------------------------------------------------------------------------------------------------------------------							
21	---------------------------------------------------------------------------------------------------------------------------------------------------							
22	539.98 ± 5.66	3.0061 ± 0.10	3240.04 ± 131.65	3043.45 ± 105.8	2949.38 ± 104.14	1717.18 ± 63.52	231.11 ± 7.98	0.177
23	623.35 ± 22.77	3.0344 ± 0.07	3830.39 ± 23.96	3596.49 ± 120.4	3483.29 ± 78.06	2178.92 ± 86.02	221.04 ± 8.00	0.331
24	581.92 ± 15.64	3.5004 ± 0.07	3281.42 ± 41.15	3092.56 ± 40.03	2985.90 ± 56.34	1190.76 ± 73.31	183.69 ± 8.38	0.286
25	544.78 ± 7.02	2.9202 ± 0.07	3190.04 ± 140.05	2972.93 ± 84.97	2868.78 ± 145.87	963.75 ± 10.88	222.32 ± 12.29	0.178
26	544.50 ± 9.80	3.1049 ± 0.06	3355.88 ± 86.63	2993.48 ±110.66	2858.42 ± 80.39	1353.17 ± 74.18	218.83 ± 7.87	0.158
27	680.23 ± 18.60	3.8571 ± 0.14	3360.59 ± 79.81	3169.73 ± 89.50	3057.67 ± 121.22	1826.29 ± 24.42	234.47 ±5.50	0.237

**Table 7:** Mechanical and thermomechanical characterization results for TPU 95A specimens. Values are reported as mean ± standard deviation from three independent repetitions.

Experiment No.	Output Variables for TPU 95A						
	F_max_ (N)	Def_max_ (mm)	E′_25°C_ [MPa]	E′_75°C_ [MPa]	E′_100°C_ [MPa]	E′_150°C_ [MPa]	Tan δ
1	122.26 ± 2.86	104.39 ± 3.12	188.50 ± 5.79	85.810 ± 1.95	56.820 ± 3.37	13.700 ± 0.07	0.137
2	379.62 ± 3.67	111.08 ± 1.36	181.32 ± 3.01	78.399 ± 1.84	50.632 ± 0.46	17.626 ± 0.26	0.161
3	204.12 ± 4.57	124.45 ± 3.18	147.04 ± 2.99	48.604 ± 1.43	33.345 ± 0.68	9.984 ± 0.02	0.134
4	228.87 ± 0.52	188.34 ± 1.59	144.30 ± 4.39	58.359 ± 1.79	36.814 ± 0.54	36.814 ± 0.21	0.188
5	322.77 ± 4.42	205.97 ± 9.93	155.36 ± 3.24	51.897 ± 0.24	31.203 ± 0.66	11.083 ± 0.46	0.214
6	325.23 ± 0.78	180.54 ± 8.37	201.39 ± 7.56	77.212 ± 1.05	54.259 ± 0.68	23.289 ± 0.32	0.151
7	176.38 ± 3.53	156.28 ± 5.60	118.74 ± 0.26	51.136 ± 1.92	36.535 ± 0.59	12.770 ± 0.58	0.178
8	231.48 ± 3.00	165.31 ± 0.53	121.32 ± 1.33	44.660 ± 0.40	30.380 ± 0.40	10.250 ± 0.56	0.228
9	227.37 ± 2.58	114.19 ± 1.07	151.35 ± 0.48	57.572 ± 1.79	41.220 ± 1.85	12.760 ± 0.32	0.278
10	198.12 ± 2.02	158.37 ± 1.32	117.54 ± 4.47	50.804 ± 0.80	32.649 ± 1.35	7.195 ± 0.34	0.206
11	141.33 ± 4.11	135.09 ± 5.66	113.52 ± 2.55	34.692 ± 0.94	19.568 ± 1.14	2.188 ± 0.04	0.150
12	247.05 ± 0.27	166.35 ± 8.04	118.68 ± 4.04	45.525 ± 1.35	24.798 ± 0.77	12.131 ± 0.72	0.221
13	183.59 ± 2.24	142.52 ± 4.88	100.59 ± 3.43	41.279 ± 0.05	26.219 ± 0.67	6.857 ± 0.37	0.206
14	216.99 ± 5.47	179.56 ± 5.82	120.68 ± 3.41	52.804 ± 0.50	33.572 ± 0.23	8.968 ± 0.49	0.188
15	336.69 ± 5.55	199.03 ± 8.35	169.23 ± 3.47	65.596 ± 2.30	40.577 ± 2.24	7.370 ± 0.02	0.259
16	225.49 ± 5.74	173.78 ± 8.07	129.17 ± 3.33	50.895 ± 0.24	31.281 ± 0.20	5.691 ± 0.24	0.137
17	284.65 ± 1.37	191.20 ± 4.01	124.02 ± 2.22	45.815 ± 1.02	27.520 ± 0.99	13.284 ± 0.16	0.210
18	271.93 ± 5.59	223.03 ± 7.86	168.35 ± 3.28	62.916 ± 0.20	45.188 ± 1.50	17.676 ± 0.82	0.139
19	200.30 ± 1.24	154.49 ± 4.99	158.92 ± 4.03	48.433 ± 0.24	25.729 ± 0.53	22.328 ± 0.83	0.264
20	155.00 ± 2.45	130.05 ± 4.40	85.57 ± 1.86	36.880 ± 0.25	18.685 ± 0.60	2.209 ± 0.04	0.237
21	147.51 ± 1.16	109.22 ± 0.39	149.72 ± 1.87	50.759 ± 1.08	30.413 ± 0.10	10.016 ± 0.46	0.131
22	143.09 ± 1.20	129.62 ± 1.18	144.43 ± 1.85	48.651 ± 1.20	24.880 ± 1.32	2.793 ± 0.12	0.201
23	220.94 ± 5.62	177.00 ± 1.08	142.29 ± 3.98	58.296 ± 1.63	35.134 ± 1.49	6.893 ± 0.28	0.190
24	153.28 ± 2.99	128.44 ± 3.43	135.18 ± 2.65	47.772 ± 0.57	30.975 ± 0.95	5.780 ± 0.27	0.221
25	188.54 ± 4.13	140.14 ± 5.72	149.83 ± 0.34	62.347 ± 1.81	39.548 ± 1.56	7.773 ± 0.09	0.202
26	204.89 ± 1.51	165.14 ± 5.90	134.06 ± 2.33	48.957 ± 1.31	30.909 ± 0.11	4.829 ± 0.25	0.136
27	239.55 ± 3.00	168.45 ± 8.13	128.24 ± 2.02	52.549 ± 1.11	34.187 ± 1.93	8.052 ± 0.03	0.210

**Table 8:** ANOVA results for TPU 95A.

Response	Parameter	DF (Degrees of Freedom)	SS (Sum of Squares)	MS (Mean Square)	F (F-Value)	*p* (*p*-Value)	% Contrib
F_max_ (N)	ET (°C)	2	27,030.0	13,515.3	4.5399	0.0263	27.05
	LH (mm)	2	3344.01	1672.00	0.5616	0.5805	3.35
	PT (°C)	2	7007.78	3503.89	1.1770	0.3321	7.01
	PS (mm/s)	2	11,934.2	5967.13	2.0044	0.1654	11.94
Def_max_ (mm)	ET (°C)	2	4020.54	2010.27	2.6248	0.1015	17.27
	LH (mm)	2	5773.79	2886.90	3.7694	0.0442	24.8
	PT (°C)	2	250.643	125.322	0.1636	0.8504	1.08
	PS (mm/s)	2	220.257	110.129	0.1438	0.8671	0.95
Tan δ	ET (°C)	2	0.0009	0.0005	0.1820	0.8352	1.92
	LH (mm)	2	0.0022	0.0011	0.4480	0.6463	4.72
	PT (°C)	2	0.0017	0.0009	0.3491	0.7103	3.68
	PS (mm/s)	2	0.0001	0.0000	0.0189	0.9813	0.20
E_25°C_ (MPa)	ET (°C)	2	2421.90	1210.90	2.2252	0.1385	15.11
	LH (mm)	2	704.516	352.258	0.6473	0.5359	4.40
	PT (°C)	2	1215.90	607.947	1.1172	0.3501	7.59
	PS (mm/s)	2	2435.20	1217.60	2.2376	0.1372	15.19
E_75°C_ (MPa)	ET (°C)	2	373.788	186.894	2.2199	0.1391	13.93
	LH (mm)	2	179.456	89.7280	1.0658	0.3664	6.69
	PT (°C)	2	504.487	252.244	2.9961	0.0768	18.8
	PS (mm/s)	2	194.773	97.3867	1.1567	0.3381	7.26
E_100°C_ (MPa)	ET (°C)	2	420.160	210.080	4.5950	0.0254	23.53
	LH (mm)	2	170.822	85.4113	1.8682	0.1848	9.57
	PT (°C)	2	204.947	102.473	2.2414	0.1368	11.48
	PS (mm/s)	2	212.714	106.357	2.3263	0.1279	11.91
E_150°C_ (MPa)	ET (°C)	2	394.881	197.441	3.8424	0.0420	27.23
	LH (mm)	2	19.2800	9.6400	0.1876	0.8306	1.33
	PT (°C)	2	77.8044	38.9022	0.7571	0.4842	5.37
	PS (mm/s)	2	84.6552	42.3276	0.8237	0.4556	5.84

**Table 9:** ANOVA results for Z-PEEK.

Response	Parameter	DF (Degrees of Freedom)	SS (Sum of Squares)	MS (Mean Square)	F (F-Value)	*p* (*p*-Value)	% Contrib
F_max_ (N)	ET (°C)	2	2312.45	1156.22	0.4981	0.6181	4.0129
	PT (°C)	2	15,756.8	7878.39	3.3937	0.0628	27.344
	CT (°C)	2	7055.64	3527.82	1.5197	0.2528	12.244
Def_max_ (mm)	ET (°C)	2	0.3631	0.1816	1.5158	0.2536	12.622
	PT (°C)	2	0.5936	0.2968	2.4778	0.1199	20.632
	CT (°C)	2	0.2434	0.1217	1.0160	0.3872	8.4602
Tan δ	ET (°C)	2	0.0180	0.0090	1.6348	0.2301	12.710
	PT (°C)	2	0.0294	0.0147	2.6668	0.1044	20.734
	CT (°C)	2	0.0172	0.0086	1.5604	0.2446	12.132
E_25°C_ (MPa)	ET (°C)	2	841,111	420,555	1.8964	0.1867	16.879
	PT (°C)	2	570,083	128,541	0.5796	0.5729	5.1588
	CT (°C)	2	780,445	390,222	1.7596	0.2081	15.661
E_75°C_ (MPa)	ET (°C)	2	681,758	340,879	1.6399	0.2291	15.216
	PT (°C)	2	249,867	124,933	0.6011	0.5618	5.5768
	CT (°C)	2	638,851	319,425	1.5367	0.2492	14.259
E_100°C_ (MPa)	ET (°C)	2	698,782	349,391	1.7997	0.2015	16.447
	PT (°C)	2	232,858	116,429	0.5997	0.5625	5.4807
	CT (°C)	2	599,113	299,556	1.5430	0.2479	14.101
E_150°C_ (MPa)	ET (°C)	2	103,853	51,936.6	1.5423	0.2481	13.404
	PT (°C)	2	20,299.3	10,149.6	0.0301	0.9704	0.2619
	CT (°C)	2	1,975,723	987,861	2.9337	0.0863	25.497
E_200°C_ (MPa)	ET (°C)	2	71.8406	35.9203	0.0123	0.9877	0.1261
	PT (°C)	2	3870.90	1935.45	0.6660	0.5293	6.7979
	CT (°C)	2	12,325.6	6162.83	2.1208	0.1568	21.643

**Table 10:** EWM Analysis results.

Criterion	Abbreviation	Z-PEEK (*n* = 21)					TPU 95 A (*n* = 27)			
		F_max_	Def_max_	E′_25°C_	E′_75°C_		F_max_	Def_max_	E′_25°C_	E′_75°C_
Entropy	$E_{j}$	0.9987	0.9981	0.9967	0.9967		0.9888	0.9945	0.9951	0.9945
Weight	$w_{j}$	0.1304	0.1960	0.3326	0.3410		0.4129	0.2033	0.1808	0.2030

**Table 11:** VIKOR method applied to Z-PEEK. Rows marked with “---” correspond to experimental combinations that could not be manufactured because CT > PT, as explained in Section 2.2.1 and Table 3.

Exp. No	F_max_ (N)	Def_max_ (mm)	E′_25°C_ [MPa]	E′_75°C_ [MPa]	$S_{j}$	Rank $S_{j}$	$R_{i}$	Rank $R_{i}$	$Q_{i}$	Rank $Q_{i}$
1	559.06	3.5136	3947.72	3724.66	0.3543	5	0.0979	4	0.2009	5
2	---------------------------------------------------------------------------------------------------------------------------------------------------									
3	---------------------------------------------------------------------------------------------------------------------------------------------------									
4	573.94	2.967	3203.77	3024.61	0.5408	10	0.2167	13	0.5351	12
5	543.17	3.4012	3481.75	3278.33	0.5160	8	0.1686	7	0.4295	7
6	522.74	3.7417	2813.59	2656.11	0.8060	20	0.2864	20	0.8223	20
7	644.06	3.7781	3317.20	3169.42	0.5454	11	0.1916	10	0.4903	10
8	641.27	4.2931	2952.83	2793.72	0.7464	19	0.2604	19	0.7381	19
9	587.31	3.5044	3024.81	2807.95	0.6679	17	0.2577	18	0.6871	18
10	498.40	3.7606	2503.70	2367.80	0.9330	21	0.3410	21	1.0000	21
11	---------------------------------------------------------------------------------------------------------------------------------------------------									
12	---------------------------------------------------------------------------------------------------------------------------------------------------									
13	561.59	2.7360	4256.53	4042.95	0.1409	2	0.0883	2	0.0582	2
14	605.77	3.4479	4346.45	4009.49	0.1919	3	0.0896	3	0.0904	3
15	608.92	3.2878	3295.03	2980.52	0.5467	12	0.2250	15	0.5543	15
16	607.54	3.2193	3394.70	3190.22	0.4820	7	0.1853	8	0.4413	8
17	694.21	3.3499	4422.92	4168.51	0.0773	1	0.0773	1	0.0000	1
18	671.93	3.4982	3761.95	3481.33	0.3554	6	0.1301	6	0.2628	6
19	616.29	3.8298	3123.13	2822.87	0.6697	18	0.2549	17	0.6828	17
20	---------------------------------------------------------------------------------------------------------------------------------------------------									
21	---------------------------------------------------------------------------------------------------------------------------------------------------									
22	539.98	3.0061	3240.04	3043.45	0.5548	14	0.2131	12	0.5365	13
23	623.35	3.0344	3830.39	3596.49	0.2958	4	0.1083	5	0.1866	4
24	581.92	3.5004	3281.42	3092.56	0.5726	16	0.2038	11	0.5292	11
25	544.78	2.9202	3190.04	2972.93	0.5628	15	0.2264	16	0.5665	16
26	544.50	3.1049	3355.88	2993.48	0.5536	13	0.2225	14	0.5537	14
27	680.23	3.8571	3360.59	3169.73	0.5237	9	0.1892	9	0.4729	9
$f_{i}^{*}$	694.21	2.7360	4422.92	4168.51						
$f_{i}^{-}$	498.40	4.2931	2503.70	2367.80						

**Table 12:** VIKOR method applied to TPU 95A.

Exp. No	F_max_ (N)	Def_max_ (mm)	E′_25°C_ [MPa]	E′_75°C_ [MPa]	$S_{j}$	Rank $S_{j}$	$R_{i}$	Rank $R_{i}$	$Q_{i}$	Rank $Q_{i}$
1	122.26	104.39	188.50	85.81	0.4331	4	0.4129	27	0.7551	20
2	379.62	111.08	181.32	78.40	0.0722	1	0.0313	1	0.0000	1
3	204.12	124.45	147.04	48.60	0.5485	9	0.2816	16	0.6646	12
4	228.87	188.34	144.30	58.36	0.5838	13	0.2419	10	0.6376	10
5	322.77	205.97	155.36	51.90	0.4718	6	0.1741	5	0.4695	4
6	325.23	180.54	201.39	77.21	0.2519	2	0.1305	2	0.2569	2
7	176.38	156.28	118.74	51.14	0.6817	24	0.3261	21	0.8171	21
8	231.48	165.31	121.32	44.66	0.6305	18	0.2377	9	0.6651	13
9	227.37	114.19	151.35	57.57	0.4513	5	0.2443	11	0.5470	7
10	198.12	158.37	117.54	50.80	0.6536	21	0.2912	18	0.7515	19
11	141.33	135.09	113.52	34.69	0.7751	26	0.3823	26	0.9568	27
12	247.05	166.35	118.68	45.52	0.6080	16	0.2127	7	0.6164	9
13	183.59	142.52	100.59	41.28	0.7140	25	0.3145	20	0.8248	23
14	216.99	179.56	120.68	52.80	0.6468	20	0.2609	14	0.7071	17
15	336.69	199.03	169.23	65.60	0.3615	3	0.1622	4	0.3759	3
16	225.49	173.78	129.17	50.90	0.6176	17	0.2473	12	0.6685	14
17	284.65	191.20	124.02	45.82	0.5807	11	0.1588	3	0.5265	5
18	271.93	223.03	168.35	62.92	0.5186	7	0.2033	6	0.5409	6
19	200.30	154.49	158.92	48.43	0.5883	14	0.2877	17	0.7008	16
20	155.00	130.05	85.57	36.88	0.7794	27	0.3604	22	0.9312	26
21	147.51	109.22	149.72	50.76	0.6005	15	0.3724	24	0.8204	22
22	143.09	129.62	144.43	48.65	0.6592	23	0.3795	25	0.8712	25
23	220.94	177.00	142.29	58.30	0.5805	10	0.2546	13	0.6519	11
24	153.28	128.44	135.18	47.77	0.6588	22	0.3632	23	0.8495	24
25	188.54	140.14	149.83	62.35	0.5415	8	0.3066	19	0.6924	15
26	204.89	165.14	134.06	48.96	0.6359	19	0.2804	15	0.7248	18
27	239.55	168.45	128.24	52.55	0.5808	12	0.2247	8	0.6129	8
$f_{i}^{*}$	379.62	104.39	201.39	85.81						
$f_{i}^{-}$	122.26	223.03	85.57	34.69						

## Data Availability

The original contributions presented in this study are included in the article. Further inquiries can be directed to the corresponding author.

## Abbreviations

The following abbreviations are used in this manuscript:

AM	Additive Manufacturing
ASTM	American Society for Testing and Materials
PBF	Powder Bed Fusion
DED	Directed Energy Deposition
ME	Material Extrusion
VAT	Vat Photopolymerization
BJ	Binder Jetting
MJ	Material Jetting
SL	Sheet Lamination
FFF	Fused Filament Fabrication
DoE	Design of Experiments
DMA	Dynamic Mechanical Analysis
ANOVA	Analysis of Variance
EWM	Entropy Weight Method
VIKOR	VlseKriterijumska Optimizacija I Kompromisno Resenje
Z-PEEK	Zortrax Polyether Ether Ketone
TPU	Thermoplastic Polyurethane
FDA	Food and Drug Administration
DSC	Differential Scanning Calorimetry
ET	Extrusion Temperature
PT	Plate Temperature
CT	Chamber Temperature
LH	Layer Height
PS	Printing Speed
F_max_	Maximum Tensile Force
Def_max_	Maximum Deformation
E′	Storage Modulus
tan δ	Loss Factor
T_g_	Glass Transition Temperature
MCDM	Multi-Criteria Decision-Making
AHP	Analytic Hierarchy Process
TOPSIS	Technique for Order Preference by Similarity to Ideal Solution
ARAS	Additive Ratio Assessment
E″	Loss Modulus
